# Breastfeeding at one year after birth: duration and associated factors in primiparous women in Sweden - a longitudinal study

**DOI:** 10.1186/s13006-026-00840-x

**Published:** 2026-04-11

**Authors:** Christel Johansson, Ingegerd Hildingsson, Malin Edqvist, Anna Bonnevier, Christine Rubertsson

**Affiliations:** 1 Department of Health Sciences, Medical Faculty, Lund University, Box 117, Lund, SE-221 00 Sweden; 2 Department of Obstetrics and Gynecology, Ystad Hospital, Lasarettsgatan 7A, Ystad, SE-271 82 Sweden; 3 Department of Health Science, Mid Sweden University, Sundsvall, Sweden; 4 Department of Women’s and Children’s Health, Karolinska Institutet, Solna, Sweden; 5 Department of Women’s Health & Health Professions, Karolinska University Hospital, Stockholm, Sweden; 6 Department of Clinical Sciences Lund, Lund University, Lund, Sweden; 7 Department of Obstetrics and Gynecology, Skane University Hospital, Lund, Sweden

**Keywords:** Breastfeeding, Breastfeeding duration, Infant, Newborn, Postnatal care, Primiparous women

## Abstract

**Background:**

Exclusive breastfeeding during the first six months of life is a global public health recommendation and offers well-established and significant benefits for infant health and development, as well as health benefits for women. Most breastfeeding research focuses on the early postpartum period, despite the WHO recommendations to continue breastfeeding for up to two years or longer. Investigating factors associated with breastfeeding at one year may inform healthcare practices aimed at supporting women to sustain breastfeeding over time. Therefore, the aim of this study was to explore breastfeeding duration and factors associated with breastfeeding at one year after birth, in primiparous women given birth vaginally.

**Methods:**

This study is a longitudinal cohort based on follow-up data from a multicentre randomized controlled trial at five obstetric units in Sweden. Data were derived from questionnaires sent to women at one month and one year postpartum. Multivariable logistic regression analysis was used to analyze factors associated with breastfeeding at one year after birth, and Kaplan-Meier analysis estimated breastfeeding duration.

**Results:**

The prevalence of breastfeeding at one year after birth was 31% in this cohort of 1739 women who gave birth vaginally to their first child. The mean duration of breastfeeding was 5.62 months. Exclusive breastfeeding during the first four months (aOR; 2.85; 2.25–3.62), not having Swedish as native language (aOR; 1.69; 1.30–2.18), experiencing a very positive first breastfeeding session (aOR; 1.67; 1.34–2.08) and having a university education (aOR; 1.53 1.17–1.99), were associated with breastfeeding at one year.

**Conclusions:**

This study highlights the importance of providing breastfeeding support to ensure a very positive first breastfeeding experience, avoiding introduction of formula or solids before four months of age unless medically indicated and including social background factors. Knowledge about factors associated with breastfeeding at one year can inform healthcare interventions aiming to increase breastfeeding in line with national and international recommendations. These factors should be implemented in future breastfeeding support strategies.

## Background

Breastmilk is the optimal source of nutrition for infants, with a composition that adapts to the infants’ age and needs. Breastfeeding is associated with positive health outcomes, offering both short- and long-term benefits for infants and women, as well as economic and environmental benefits for society [1–6]. For many health outcomes, the protective effects of breastfeeding appear to increase with a longer duration of breastfeeding; each additional month of breastfeeding may confer incremental benefits for both maternal and child health although no specific optimal length of breastfeeding has been identified [4]. Nevertheless, international guidelines recommend exclusive breastfeeding for the first six months of life and continued breastfeeding up to two years of age or beyond [7]. In addition, breastfeeding has environmental advantages compared to infant formula. As breastmilk is the natural and sustainable way to feed the infant, it is important to protect, support and promote breastfeeding and its health benefits [2]. Due to these significant benefits, the World Health Organization (WHO) has set a global nutrition target to increase the rate of exclusive breastfeeding during the first six months to at least 50% by 2025 [7, 8]. In Sweden, national infant feeding recommendations to a high extent align with those of the WHO, with the exception that continued breastfeeding is recommended for up to one year, or for as long as mother and child desire [9]. Despite the known health benefits, breastfeeding rates drop steeply soon after birth [10]. Prelacteal feeds (the practice of giving newborns substances other than breast milk during the first days of life) are strongly associated with delayed initiation of breastfeeding [11]. Self-reported insufficient milk production is a common reason for introducing formula which often leads to breastfeeding cessation, often reinforced by parental and professional misinterpretation of normal infant behaviors as signs of milk inadequacy [11]. In Sweden, early supplementation with formula has become increasingly common. Exclusive breastfeeding at one week declined from 94% in 1997 to 72% in 2021, while partial breastfeeding increased from 5% to 21% [10]. These trends highlight the challenges women face in adhering to WHO’s recommendation of six months of exclusive breastfeeding. Moreover, the prevalence of exclusive breastfeeding for six months has decreased over the last few decades, reaching only 12% in 2021. In contrast, breastfeeding beyond six months has increased since 2010; among twelve-month-old infants, rates rose from 16% in 2010 to 30% in 2021 [10]. In addition, Sweden’s national recommendations conflict with those of the WHO guidelines. Since 2011, the National Food Agency has advised parents that small portions of solid food (1mL) can be introduced from four months of age [12]. This guidance may influence both the timing and continuation of exclusive breastfeeding, as shown in a Swedish study where a higher age at the introduction of small tasting portions was associated with a longer duration of breastfeeding [13]. Another factor identified in Swedish research includes longer total parental leave, reflecting the combined leave of both parents, which has been shown to support longer duration of breastfeeding [14]. Prior reviews found that not smoking, having a vaginal birth, high maternal educational attainment, and specific breastfeeding education were associated with higher rates of breastfeeding duration [15]. Swedish studies highlight the crucial role of healthcare professionals; both midwives and child health care nurses (CHCNs´) in supporting breastfeeding. Women emphasize the importance of sensitive and individualized guidance to promote a positive breastfeeding experience [16].

The period immediately following birth, here referred to as the *initial postnatal period*, encompasses the first two hours of the infant’s life and the care provided during this time, referred to as *initial postnatal care*. This phase encompasses several critical events during which the midwife holds a central responsibility for monitoring, assessing, and taking appropriate actions related to maternal and neonatal morbidity and mortality, as well as updating care plans accordingly. Key aspects include support for the mother–infant dyad through uninterrupted skin-to-skin contact and initiation of breastfeeding [17, 18]. The infant´s respiratory and cardiovascular adaptation and overall adaptation is assessed using the Apgar-score screening [19]. Clinical management further includes facilitating the birth of the placenta, ensuring adequate uterine contraction to prevent excessive postpartum hemorrhage [17] and the examination, accurate diagnosis and repair of perineal trauma [20].

Most research on breastfeeding focuses on the early postpartum period, while breastfeeding beyond the first year remains relatively understudied, despite WHO recommendations to continue for up to two years or more. This knowledge gap highlights the need for more information on how long exclusive and partial breastfeeding are practised in Sweden and which factors influence breastfeeding duration (Nordic Nutrition Recommendations, 2023) [21]. Investigating factors associated with breastfeeding at one year is important for improving breastfeeding duration. Therefore, the aim of this longitudinal study was to explore breastfeeding duration and factors associated with breastfeeding at one year after birth, in primiparous women given birth vaginally.

## Methods

### Design

This longitudinal cohort study is a secondary analysis of a follow-up questionnaire sent to participants in a multicentre randomized trial [22] aimed at evaluating a collegial midwifery intervention to reduce perineal trauma. The full details of the trial have been described elsewhere [23]. The trial was conducted between 2018 and 2020, with follow-up questionnaires sent to participating women between 2019 and 2022.

### Setting

The trial was conducted at five obstetric units in different parts of Sweden: four university hospitals and one county hospital, with an annual number of births ranging from 2800 to 5200. In Sweden, midwives provide postnatal care for the mother and infant during the first week, either in hospital or through home-based postnatal midwifery care [24]. From the second week onward, families are referred to a Child Health Care Nurse (CHCN) at Well-Baby Clinics (WBCs), who offer guidance on breastfeeding and overall child health within a national program for children aged 0–6 years [25].

### Participants

The inclusion criteria for this study were participation in the RCT, which included primiparous women who had a vaginal non-instrumental birth, at ≥ 37 + 0 weeks of gestation, with a singleton live fetus in vertex presentation. Furthermore, participants were required to have completed two follow-up questionnaires, administered one month and one year postpartum.

### Data collection

The questionnaires were developed to evaluate the outcomes of interest related to the trial, including breastfeeding. The items were developed by researchers with extensive and well-established experience in the field and were reviewed for face validity by women with recent childbirth experience. To assess the validity and relevance of the questionnaire items, ten women who had recently given birth were invited to review the questionnaire using a think-aloud process with cognitive interviewing. Women found the questions relevant and understandable, and only minor adjustments were made with regard to wording. The questionnaires consisted of self-reported data and were completed by the participants at one month and one year postpartum. In addition to questionnaire data collected at one month and one year postpartum, background data were obtained from medical records. Data from the medical records which pertained to the woman included ethnicity, body mass index (BMI) at first booking visit at antenatal clinic, onset of labor (spontaneous or induction), epidural analgesia, postpartum haemorrhage, third- or fourth-degree tears, i.e., severe perineal tears and episiotomy. Data about newborn health; Apgar and admission to neonatal intensive care unit (NICU) were also collected from medical records.

The questionnaires included questions concerning sociodemographic background, i.e., marital status, level of education, native language, employment and tobacco use. Other variables such as Edinburgh postnatal depression scale (EPDS) [26, 27] at one month and one year postpartum, fear of birth during pregnancy (FOBS) [28], history of mental health, the global birth experience [29] and a question about self-rated health [30] were also investigated. The breastfeeding initiation was assessed at one month postpartum, as well as the questions regarding breastfeeding support from healthcare professionals and women’s experience of the first breastfeeding session. At the one-year follow-up, women were asked about the timing of introduction of foods or liquids other than breastmilk and whether they were still breastfeeding. The questionnaires were available in Swedish and English. Oral and written information was provided, and written informed consent was obtained. The Swedish version was available both electronically and in print, whereas the English version was available only in print. Women who provided an e-mail address received a link to the web-based questionnaire, while those without an e-mail address, as well as those who did not respond to the digital version, were sent a printed copy by post. Up to four reminders were sent for each questionnaire.

### Outcome and explanatory variables

The outcome variable was breastfeeding at one year after birth, defined as any breastfeeding (exclusive or partial), and measured by the question: *“Do you breastfeed your child (when it was one year old)?”* Yes/No. In addition, women were asked about breastfeeding cessation (in months) using the question *“How old was your child when you stopped breastfeeding?”.*

The explanatory variables included background variables, obstetric and newborn variables and first breastfeeding and health variables at one year after birth. The continuous variables were categorized as follows; maternal age: <25 years, 25–35 years, > 35 years, BMI according to the WHO classification: <18.5, 18.5–24.9, 25.0-29.9, $\:\ge\:$30, and post-partum haemorrhage: <1000 ml/>1000 ml or more. *Marital status* was assessed using a categorical question with the response options: ‘Married/living with a partner’, ‘Have a partner but do not live together’, ‘Single’ and ‘Other (please specify)’. For analysis, the variable was dichotomized into ‘Married or living with a partner’ (ref) versus ‘Not living with a partner or other life situation’. *Level of education* was assessed on a 4-point ordinal scale with the response alternatives: ‘Primary school’, ‘Secondary school’, ‘University education 1–3 years’ and ‘University education over 3 years’. The variable was then dichotomized into ‘University education’ (ref) versus ‘No university education’. Following questions “*Do you smoke?*”, “*Did you experience fear of childbirth before giving birth?*” and “*Did you have any history of mental health issues before pregnancy?*” were dichotomized and coded as ‘Yes’/‘No’. Questions about the first breastfeeding session in one month postpartum questionnaire were as follow: “*When did you breastfeed for the first time?*” with the response options: ‘Immediately after giving birth (within two hours after birth)’, ‘More than two hours after birth’, ‘Other, please describe’ and ‘Did not start breastfeeding’. The variable was then dichotomized into ‘Breastfeeding within two hours’ (ref) versus ‘Breastfeeding after more than two hours’. *“Was your newborn kept skin-to-skin with you until the first breastfeeding session and/or until asleep?”* with the response options: ‘Yes’, ‘No, it was disrupted because of care giving routines for the newborn’, ‘No, it was disrupted because of care giving routines for myself’, ‘No, I did not want skin to skin care’, ‘No, my partner gave skin to skin care’, ‘No, the caregivers wanted to measure and weigh the baby’ and ‘Can´t recall’. The variable was then dichotomized into ‘Uninterrupted initial skin-to-skin care’ (ref) versus ‘No or disrupted initial skin-to-skin care’. “*Did you get the support you needed during the first breastfeeding session*?” was answered with ‘Yes’ or ‘No’ options, with ‘Yes’ serving as the reference group. The question “*How old was your child when you first fed him/her something other than breast milk?”* had response options for each month up to six months and was used to identify the timing of introduction of complementary feeding. To perform the analysis, the variable was dichotomized using a cut-off at four months [13]. For the question “*How old was your child at the time of the cessation of breastfeeding?”* was assessed on a continuous variable. The question *“How did you experience the first breastfeeding session?”* was on a 4-point Likert scale ‘Very positive’, ‘Positive’, ‘Negative’ and ‘Very Negative’ with responses categorized as ‘Very positive’ (ref) versus ‘less than very positive’ (ref). The question *Employment at one year* was assessed using a categorical question with the response options: ‘Employed or self-employed’, ‘Student’, ‘Maternity leave’, ‘Unemployed’, and ‘Other (please specify)’. For analysis, ‘Employed’ and ‘Student’ were combined as the reference category, while ‘Maternity leave’ and ‘Unemployed’ were kept as separate groups. The variable *Self-rated current health* was measured using a 5-point Likert scale and subsequently dichotomized as ‘Very good and good’ versus ‘Less than good’. The selection of explanatory variables was based on previous research about breastfeeding and clinical reasoning.

### Statistical analysis

Descriptive statistics were used to present the background characteristics and the initial breastfeeding session. Women´s characteristics were described using means (standard deviations, SD) for continuous variables and numbers and percentages for categorical variables. Statistical significance was set to *P* < 0.05. Kaplan–Meier survival analysis was performed to estimate the mean duration of breastfeeding and to provide a graphical illustration of breastfeeding cessation over time.

The analysis was conducted stepwise. First, crude odds ratios (ORs) with 95% confidence intervals (CIs) were calculated for each explanatory variable separately (sociodemographic background, obstetric and neonatal variables, health and breastfeeding-related variables), using breastfeeding at one year as the outcome. All background, breastfeeding and health-related variables that were statistically significant in the crude analyses were then included in a multivariable logistic regression model. Variables that were not statistically significant in both the crude and adjusted model were systematically removed one by one based on their significance levels, resulting in the final model, Table 4. All statistical analyses were carried out using IBM SPSS Statistics version 29 (IBM, Armonk, NY, USA). The study was approved by the regional Ethics Board of Lund University, Sweden (2018/476) in July 2018.

## Results

**Fig. 1:**
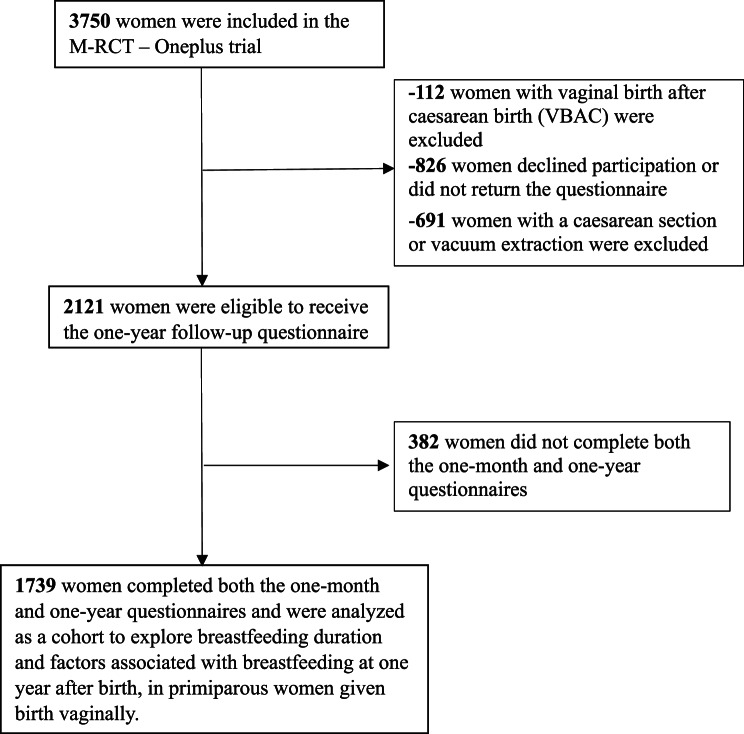
Flowchart of the women randomized in the Oneplus trial and participation in the one-month and one-year follow-up questionnaire

**Fig. 2:**
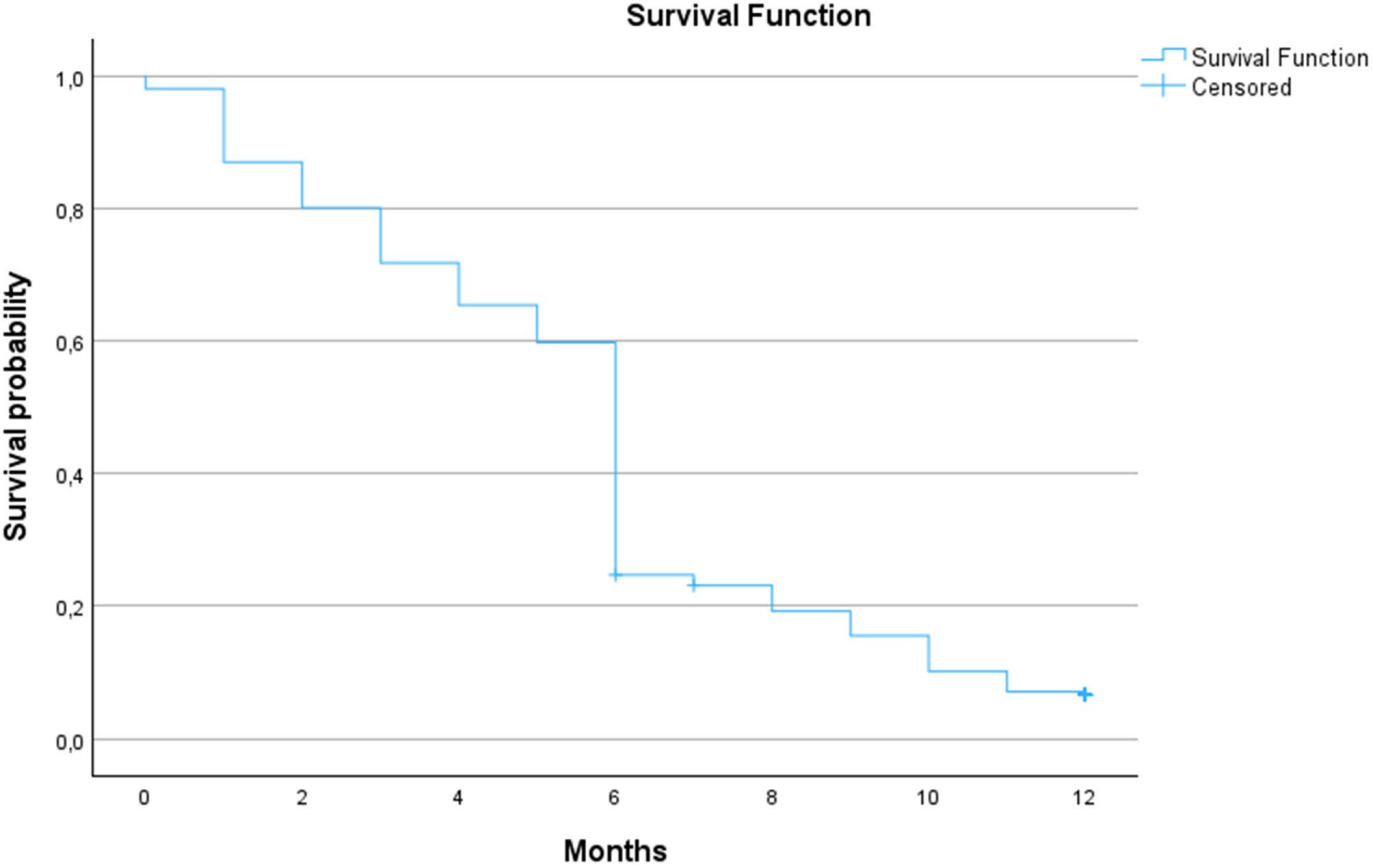
Kaplan-Meier analysis illustrates breastfeeding duration and cessation over the first year after birth

In this cohort 1739 women answered questionnaires one month and one year after birth. An overview of a flowchart is presented in Fig. 1:

The prevalence of breastfeeding at one year after birth was 31.3% (541 women). Figure 2 illustrates breastfeeding duration and cessation over the first year after birth, from 1739 who initiated breastfeeding to 541 at one year. Data on breastfeeding duration, included 1273 women in the Kaplan-Meier. The estimated mean duration of breastfeeding was 5.62 months (95% CI 5.44–5.80).

### Background characteristics

The women had a mean age of 30 years (ranging from 18 to 47) and most had Swedish as their native language (79.0%). The greater part of the population had a university education (75.3%), almost a quarter of the women reported having a history of mental health issues prior to pregnancy, and 340 women (19.1%) were identified with fear of birth. At one year postpartum, the majority were married or living with a partner (97.0%) and about half of the women were employed or studying (54.5%), slightly less than half were on maternity leave (40.5%), and few (5.0%) were unemployed. The prevalence of depressive symptoms, according to EPDS scores 12–30, remained at around 15.9% at both one month (*n* = 277) and one year (*n* = 281) postpartum. Further at one year, 87.6% of the women rated their health as very good or good. The majority of women (74.1%) had a spontaneous onset of labour, and 68.7% received epidural analgesia during birth. A small proportion experienced severe perineal trauma (3.8%). Most newborns (98.2%) were healthy at birth and did not require admission to NICU.

### First breastfeeding session

The majority (72.0%) reported initiating breastfeeding within two hours after birth. Furthermore, 78.8% reported that their newborn was kept skin-to-skin until the first breastfeeding session was established and/or until the baby fell asleep. However, some experienced separation due to caregiving routines for the baby (5.1%) or themselves (5.1%). Others reported that their partner provided skin-to-skin contact (3.6%) and 88 (5.3%) women described that healthcare providers prioritized measuring and weighing the baby. A minor proportion (2.2%) of respondents could not recall whether skin-to-skin contact was upheld. In response to the question *“Was your baby able to find the breast*,* latch on*,* and breastfeed within the first two hours after being born?”* 53.5% reported that their baby was able to do so, while 9.9% stated that their baby was not interested and 13.3% women needed to assist their baby in latching. In addition, 16.9% reported that healthcare providers used hands-on techniques for facilitating latching, 5.4% experienced separation from their baby during the first two hours and 1% could not recall. When asked about introduction of anything other than breast milk to the newborn, 22.5% of mothers reported introduction at one month or younger. At two months, an additional 5% introduced anything other than breast milk, followed by an additional 7.3% at three months and 38.8% at four months. At five months, a further 12.4% introduced formula or solids, while 11.4% waited until six months or later. In total, 2.5% of respondents stated that they had never breastfed.

Table 1 shows the background variables in relation to breastfeeding at one year after birth. Older age (OR 1.39; 1.01–1.91), higher education (OR 1.47; 1.15–1.89) and not having Swedish as the native language (OR 1.96; 1.52–2.49) were associated with breastfeeding at one year.

**Table 1:** Background variables in relation to breastfeeding at one year after birth of the 1739 women in the study†

	Breastfeeding	Breastfeeding cessation	OR (95% CI)
	*n* =541 (31.3)	*n* =1189 (68.7)	
	*n* (%)‡	*n* (%)‡	
**Age**,** years**			
<25 years	33 (6.1)	100 (8.4)	0.74 (0.49-1.11)
25-35 years	439 (81.1)	978 (82.3)	1.0 ref
>35 years	69 (12.8)	111 (9.3)	1.39 (1.01-1.91)*
Missing data	0	0	
**BMI**,** kg/m² at booking visit**			
Underweight (<18.5)	13 (2.5)	23 (2.0)	1.21 (0.60-2.41)
Optimum (18.5-24.9)	330 (64.7)	705 (62.0)	1.0 ref
Overweight (25.0-29.9)	117 (22.9)	273 (24.0)	0.92 (0.71-1.18)
Obese (≥30)	50 (9.8)	136 (12.0)	0.79 (0.55-1.11)
Missing data	31 (5.7)	52 (4.4)	
**Marital status**			
Married or living with a partner	521 (96.8)	1148 (97.0)	0.93 (0.52-1.68)
Not living with a partner or other life situation	17 (3.2)	35 (3.0)	1.0 ref
Missing data	3 (0.6)	6 (0.5)	
**Native language**			
Swedish	384 (71.1)	978 (82.8)	1.0 ref
Other than Swedish	156 (28.9)	203 (17.2)	1.96 (1.52-2.49)***
Missing data	1 (0.2)	8 (0.7)	
**Level of education**			
University education	433 (80.2)	868 (73.3)	1.47 (1.15-1.89)**
No university education	107 (19.8)	316 (26.7)	1.0 ref
Missing data	1 (0.2)	5 (0.4)	
**Tobacco use at booking visit**			
No	506 (97.9)	1115 (98.6)	0.66 (0.30-1.43)
Yes	11 (2.1)	16 (1.4)	1.0 ref
Missing data	24 (4.4)	58 (4.9)	
**Depressive symptoms at one month**			
No	450 (83.3)	1003 (84.4)	0.92 (0.70-1.22)
Yes	90 (16.7)	185 (15.6)	1.0 ref
Missing data	1 (0.2)	1 (0.1)	
**Fear of birth during pregnancy**			
No	433 (80.5)	964 (81.4)	0.94 (0.73-1.22)
Yes	105 (19.5)	220 (18.6)	1.0 ref
Missing data	3 (0.6)	5 (0.4)	
**History of mental health issues**			
No	423 (78.3)	921 (77.8)	1.03 (0.81-1.32)
Yes	117 (21.7)	263 (22.2)	1.0 ref
Missing data	1 (0.2)	5 (0.4)	

*= *p*<0.05, **=*p*<0.01, ***p=<0.001

† Nine cases had missing data on breastfeeding status and were therefore not included in analyses comparing breastfeeding and breastfeeding cessation

‡ Percentages for categories with available data are calculated based on non-missing cases and therefore sum to 100%. Missing data are presented separately as percentages of the total number of cases in each group

There were no statistically significant differences in any of the obstetrical or neonatal variables regarding breastfeeding at one year (Table 2).

**Table 2:** Obstetric and newborn variables in relation to breastfeeding at one year after birth of the 1739 women in the study†

	Breastfeeding	Breastfeeding cessation	OR (95% CI)
	*n* =541 (31.3)	*n* =1189 (68.7)	
	*n* (%)‡	*n* (%)‡	
**Maternal outcomes**			
**Onset of labour**			
Spontaneous	407 (75.2)	877 (73.8)	1.08 (0.86-1.37)
Induction	134 (24.8)	312 (26.2)	1.0 ref
Missing data	0 (0)	0 (0)	
**Epidural**			
No	227 (42.0)	454 (38.2)	1.17 (0.95-1.44)
Yes	314 (58.0)	735 (61.8)	1.0 ref
Missing data	0 (0)	0 (0)	
**Post-partum haemorrhage**			
< 1000 mL	473 (90.1)	1047 (90.2)	0.99 (0.70-1.40)
≥ 1000 mL	52 (9.9)	114 (9.8)	1.0 ref
Missing data	16 (3.0)	28 (2.4)	
**Severe perineal trauma**			
Yes	16 (3.0)	50 (4.2)	1.0 ref
No	525 (97.0)	1139 (95.8)	1.44 (0.81-2.56)
Missing data	0 (0)	0 (0)	
**Episiotomy**			
No	509 (94.1)	1115 (93.8)	1.06 (0.69-1.62)
Yes	32 (5.9)	74 (6.2)	1.0 ref
Missing data	0 (0)	0 (0)	
**Very positive birth experience 1 month postpartum**			
Yes	192 (35.6)	468 (39.6)	0.84 (0.68-1.04)
No	348 (64.4)	713 (60.4)	1.0 ref
Missing data	1 (0.2)	8 (0.7)	
**Neonatal outcomes**			
**Apgar**			
≥7 vid 5 min	537 (99.3)	1182 (99.4)	0.80 (0.23-2.73)
<7 vid 5 min	4 (0.7)	7 (0.6)	1.0 ref
Missing data	0 (0)	0 (0)	
**Admission to NICUª**			
No	528 (97.6)	1170 (98.4)	0.66 (0.32-1.35)
Yes	13 (2.4)	19 (1.6)	1.0 ref
Missing data	0 (0)	0 (0)	

*= *p*<0.05, **=*p*<0.01, ***p=<0.001

ªAdmitted to the neonatal intensive care unit (NICU) due to asphyxia, respiratory distress, meconium aspiration, or infection

† Nine cases had missing data on breastfeeding status and were therefore not included in analyses comparing breastfeeding and breastfeeding cessation

‡ Percentages for categories with available data are calculated based on non-missing cases and therefore sum to 100%. Missing data are presented separately as percentages of the total number of cases in each group

Findings from the first breastfeeding session and status of employment at one year (Table 3), indicate that several factors may facilitate breastfeeding including exclusive breastfeeding within the first four months of age (OR 2.63; 2.11–3.29), breastfeeding within two hours after birth (OR 1.51; 1.19–1.91), having a very positive initial breastfeeding experience (OR 1.66; 1.34–2.05) and being unemployed one year after birth (OR 1.94; 1.24–3.02), all of which were associated with a higher likelihood of breastfeeding at one year.

**Table 3:** Initiation of breastfeeding and health variables in relation to breastfeeding at one year after birth of the 1739 women in the study†

	Breastfeeding	Breastfeeding cessation	OR (95% CI)
	*n* =541 (31.3)	*n* =1189 (68.7)	
	*n* (%)‡	*n* (%)‡	
**First breastfeeding session**			
**Very positive initial breastfeeding experience**			
Yes	242 (46.4)	382 (34.3)	1.66 (1.34-2.05)***
No	280 (53.6)	732 (65.7)	1.0 ref
Missing data	19 (3.5)	75 (6.3)	
**Initial skin-to-skin care**			
Yes	430 (81.1)	870 (77.5)	1.25 (0.96-1.61)
No	100 (18.9)	252 (22.5)	1.0 ref
Missing data	11 (2.0)	67 (5.6)	
**Breastfeeding within 2 h**			
Yes	415 (77.0)	816 (68.9)	1.51 (1.19-1.91)***
No	124 (23.0)	368 (31.1)	1.0 ref
Missing data	2 (0.4)	5 (0.4)	
**Optimal breastfeeding support from HCP**			
Yes	419 (82.2)	872 (79.9)	1.16 (0.89-1.52)
No	91 (17.8)	219 (20.1)	1.0 ref
Missing data	31 (5.7)	98 (8.2)	
**Exclusive breastfeeding for up to four months**			
Yes	215 (39.7)	238 (20.0)	2.63 (2.11-3.29)***
No	326 (60.3)	950 (80.0)	1.0 ref
Missing data	0 (0)	1 (0.1)	
**Status of employment and health related variables at one year**			
**Employment**			
Employed/Student	278 (51.5)	662 (55.7)	1.0 ref
Maternity leave	223 (41.3)	479 (40.3)	1.11 (0.90-1.37)
Unemployed	39 (7.2)	48 (4.0)	1.94 (1.24-3.02)**
Missing data	1 (0.2)	0 (0)	
**Depressive symtoms**			
No	440 (81.5)	1008 (84.8)	0.92 (0.70-1.22)
Yes	100 (18.5)	181 (15.2)	1.0 ref
Missing data	1 (0.2)	0 (0)	
**Self-rated current health good or very good**			
Yes	466 (86.1)	1051 (88.5)	0.81 (0.60-1.10)
No	75 (13.9)	137 (11.5)	1.0 ref
Missing data	0 (0)	1 (0.1)	

*= *p*<0.05, **=*p*<0.01, ***p=<0.001

† Nine cases had missing data on breastfeeding status and were therefore not included in analyses comparing breastfeeding and breastfeeding cessation

‡ Percentages for categories with available data are calculated based on non-missing cases and therefore sum to 100%. Missing data are presented separately as percentages of the total number of cases in each group

In the final model (Model 3, Table 4), several factors remained associated with breastfeeding at one year. Not having Swedish as native language (aOR 1.69; 1.30–2.18), university education (aOR 1.53 1.17–1.99), experiencing a very positive initial breastfeeding session (aOR 1.67; 1.34–2.08), and exclusive breastfeeding within the first four months of age (aOR 2.85; 2.25–3.62) were associated with a higher likelihood of breastfeeding at one year.

**Table 4:** Factors associated with breastfeeding at one year after birth of the 1739 women in the study†

	Model 1	Model 2	Model 3‡
	Background variables	Breastfeeding and health-related variables	Full model
	aOR (95% CI)	aOR (95% CI)	aOR (95% CI)
Not Swedish as native language	1.91 (1.50-2.43)***		1.69 (1.30-2.18)***
University education	1.42 (1.10-1.82)**		1.53 (1.17-1.99)**
Unemployed		1.80 (1.11-2.91)*	
Very positive initial breastfeeding experience		1.60 (1.28-1.99)***	1.67 (1.34-2.08)***
Exclusive breastfeeding up to 4 months		3.31 (2.61-4.19)***	2.85 (2.25-3.62)***

*= *p*<0.05, **=*p*<0.01, ***p=<0.001

† Nine cases had missing data on breastfeeding status and were therefore not included in analyses comparing breastfeeding and breastfeeding cessation

‡ Model 3 is adjusted for all variables retained in the final multivariable logistic regression model following stepwise selection

**Fig. 3:**
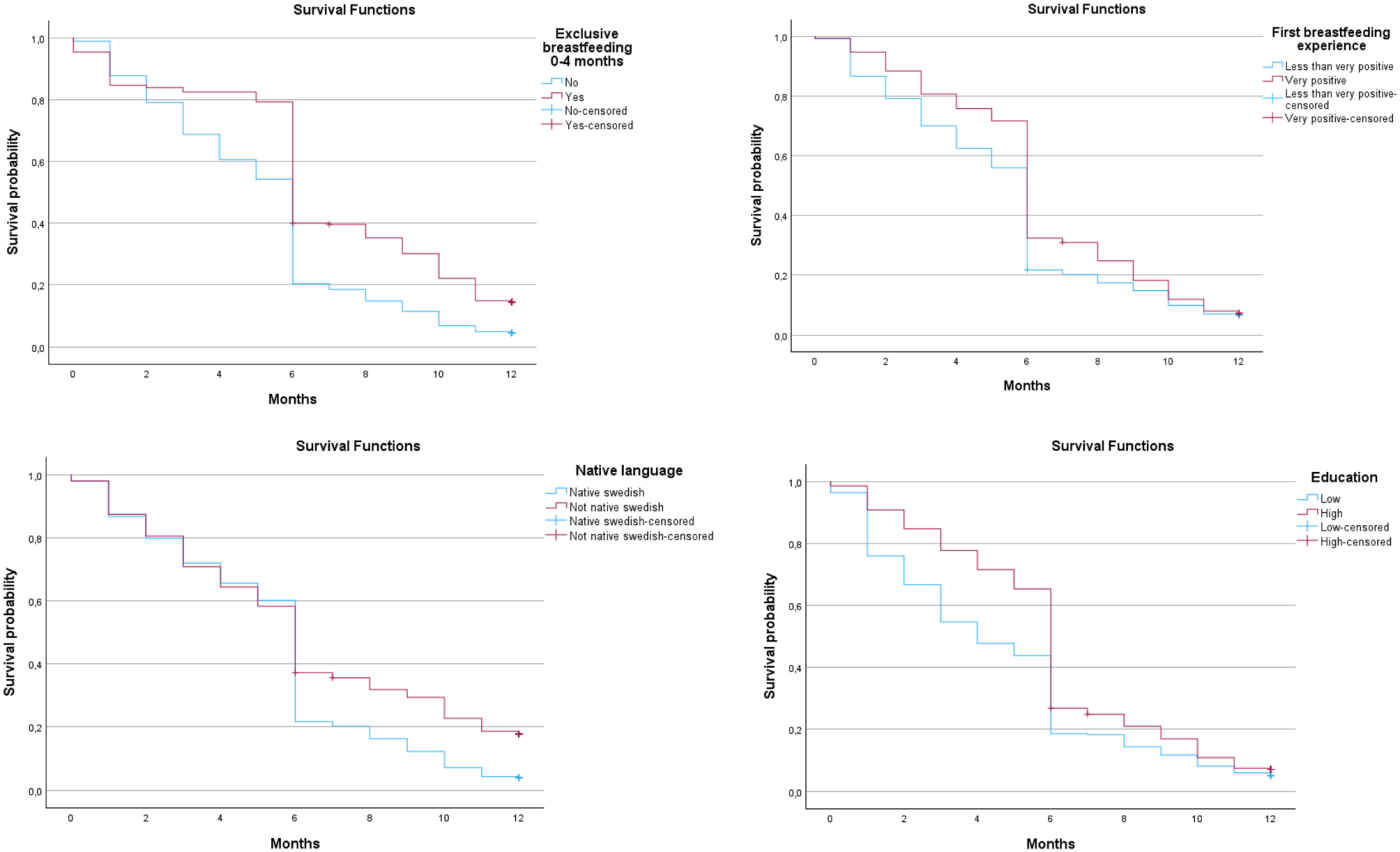
Kaplan–Meier survival estimates of breastfeeding duration and cessation during the first year after birth and stratified by four significant factors identified: exclusive breastfeeding up to four months, non-Swedish native language, a very positive initial breastfeeding experience, and maternal university education

Figure 3 illustrates breastfeeding duration during the first year, stratified by four significant factors identified in Table 4, full model: exclusive breastfeeding up to four months, non-Swedish native language, a very positive initial breastfeeding experience, and maternal university education. The Kaplan–Meier curves indicate that women who exclusively breastfed up to four months had a longer mean duration (6.90 months, 95% CI: 6.48–7.33) compared with those who did not (5.26 months, 95% CI: 5.08–5.45). Further, women who reported a very positive initial breastfeeding experience had a mean duration of 6.37 months (95% CI: 6.08–6.66) versus 5.45 months (95% CI: 5.23–5.67); women who were non-Swedish native speakers had a mean breastfeeding duration of 6.35 months (95% CI: 5.87–6.83) versus 5.44 months (95% CI: 5.26–5.62) and women with a university education had a mean duration of 5.97 months (95% CI: 5.78–6.17) versus 4.63 months (95% CI: 4.27–4.98). These results show that all four factors were associated with longer breastfeeding duration.

## Discussion

In this longitudinal cohort of primiparous women in Sweden, approximately one third were breastfeeding at one year after birth. Breastfeeding at one year was chosen as a contextually relevant outcome reflecting national recommendations and current social conditions. Several factors were associated with breastfeeding at one year, including exclusive breastfeeding up to four months and having a very positive first breastfeeding experience. In addition, certain background characteristics, such as having a university education and being a non-Swedish native, were also associated with breastfeeding at one year.

### Prevalence

The prevalence of breastfeeding at one year in our study (31.3%) corresponds with national data [10], but higher than what is commonly reported in many other high-income countries where fewer than 20% of infants are breastfed at 12 month [3, 8]. It is important to interpret these findings in relation to the context of national and global breastfeeding recommendations. While the World Health Organization recommends breastfeeding up to two years of age or beyond, Swedish infant feeding recommendations advise breastfeeding for up to one year, or for as long as the mother and child desire.

### Exclusive breastfeeding for up to 4 months

Exclusive breastfeeding up to four months of age was the strongest factor associated with a higher likelihood of breastfeeding at one year. This finding is consistent with previous research showing that early infant feeding practices are closely linked to breastfeeding duration [13, 31, 32]. Although optimal breastfeeding support from healthcare professionals was examined in the present study, it was not independently associated with breastfeeding at one year in the multivariable analysis. Nevertheless, early feeding practices are known to be influenced by clinical routines and breastfeeding support during the initial postnatal period. Previous research from Sweden has demonstrated that early introduction of formula or small tastings of solid foods may be associated with shorter breastfeeding duration, highlighting the importance of evidence-based guidance during the early months after birth [13]. In that study, 48% of all infants (*n* = 1251) were given small tastings during the fourth month. In the present study, almost 74% of infants were given formula or other foods than breastmilk by the fourth month, representing a substantial increase, and by six months almost all infants had already been introduced. Similarly, a Norwegian study found that the introduction of complementary foods before 3.5 months was associated with a higher risk of breastfeeding cessation during the first year, whereas later introduction (≥ 5 months) was associated with a lower risk of cessation [31]. In addition, a study from England demonstrated a clear dose–response relationship, showing that the earlier solids were introduced, the shorter the breastfeeding duration tended to be [32]. These findings suggest that current infant feeding recommendations in Sweden may benefit from being reviewed or clarified, particularly regarding the timing of complementary feeding. Several possible reasons have been given why formula was introduced without medical indication on the postnatal ward such as partner involvement/shared feedings [33], parental request, perceived infant dissatisfaction and lack of breastfeeding support during the postnatal hospital stay [34]. Since conflicting advice and non-evidence-based recommendations have a negative effect on breastfeeding [7, 21] it is noteworthy that many women tended to introduce formula or solids in the month preceding the recommended age. The coexistence of differing recommendations, along with variations in clinical practice, may contribute to uncertainty among both parents and healthcare professionals. The results highlight the need to strengthen breastfeeding support and care routines for mothers who wish to breastfeed, and to provide guidance on soothing infants without disrupting breastfeeding [11]. These findings also emphasize the importance of reinforcing the recommendation to defer introduction of solids foods until six months in order to support sustained breastfeeding [32].

### Not Swedish as native language

Women who did not have Swedish as their native language (21%) were more likely to breastfeed at one year compared with native Swedish speakers. International research, including a systematic review and meta-analysis, has shown similar results; immigrant women tend to breastfeed longer than native-born women [35]. A Swedish study from 2009 similarly found that mothers born outside Sweden were more likely to breastfeed at 12 months, even after adjusting for socioeconomic factors [36]. That study also reported an association between maternal income and breastfeeding at six months, indicating that both cultural and socioeconomic factors influence breastfeeding duration. In the present study, maternal income was not examined, but a high proportion of the non-Swedish-native women were highly educated (81%), which may contribute to longer breastfeeding through greater knowledge and awareness of its benefits. These findings underscore the complex interplay of cultural norms, education, and socioeconomic background in shaping breastfeeding practices in Sweden and may also reflect patterns in who participates in surveys of this kind.

### Very positive initial breastfeeding experience

Experiencing a very positive initial breastfeeding session had a significant impact on breastfeeding outcomes at one year. The findings may indicate that sensitive, responsive and evidence based support that helps women recognize and respond to their infants’ breastfeeding cues is crucial for successful early breastfeeding. Previous research in the Nordic countries has examined breastfeeding at one year postpartum [1], although few studies have provided a comprehensive analysis of the range of associated maternal, infant, and care-related factors. A systematic review and meta-analysis investigating factors associated with breastfeeding initiation and continuation (ranging from one month up to one year, although most studies focused on the first 2–4 months) found that a positive mother-infant dyad, particularly involving skin-to-skin contact after birth, was associated with higher rates of both initiation and continuation of breastfeeding [15]. Consistent with previous research, our study confirms that a very positive experience of the first breastfeeding session, characterized by initial skin-to-skin contact [18], initiation of breastfeeding within two hours, and receiving optimal breastfeeding support plays an important role [37]. Early, sensitive breastfeeding counselling and support have also been shown to increase breastfeeding rates up to six months of age [38]. In line with this evidence, our study demonstrated that women who experienced a very positive first breastfeeding session were more likely to continue breastfeeding up to one year. These findings highlight the importance of individualized, high-quality, and evidence based breastfeeding support during the initial postnatal period.

### University education

Women who were university-educated, which in our study is considered highly educated, were more likely to breastfeed at one year compared with women with no university education (lower education). This finding is consistent with previous research and supports earlier evidence on the association between maternal education and breastfeeding duration [36, 39, 40]. The results show that educated mothers tend to breastfeed for longer, which may be explained by greater knowledge of breastfeeding and its health benefits. In the present study, a majority of the women initiated both initial skin-to-skin contact with their infant and breastfed within two hours after birth, in line with WHO’s Baby-Friendly Hospital Initiative recommendations for immediate and uninterrupted skin-to-skin contact and support to initiate breastfeeding as soon as possible after birth (step 4) [37]. Slightly more than half of the women reported that their infant was able to find the breast and latch independently, while 17% required hands-on support from healthcare staff. This proportion appears to have decreased compared to a Swedish study from 2014, in which approximately 38% of women (*n* = 879) reported receiving a hands-on approach from healthcare professionals during the first breastfeeding session [41]. In that study, women who experienced hands-on support reported a more negative experience of their first breastfeeding. The lower proportion observed in our study may indicate a positive trend towards less invasive breastfeeding support practices and greater emphasis on maternal autonomy. Higher maternal education may contribute to these early breastfeeding practices by increasing mothers’ confidence and knowledge, enabling them to support the infant’s self-latching, to recognize when assistance is needed and to adhere to recommended early breastfeeding practices.

### Strengths and limitations

A strength of this study is the large longitudinal sample of mothers (*n* = 1739) recruited from five obstetric units across Sweden, including four university hospitals and one county hospital. This sample is estimated to represent approximately 18% of all births in Sweden during the study period, thereby enhancing generalizability of the findings and contributing to the knowledge of breastfeeding duration and its associated factors. Dichotomizing responses regarding birth experience and the first breastfeeding into “very positive” versus less than very positive enabled identification of experiences highlighting potential areas for improvement in care [42, 43]. Several limitations should be considered. The study sample should not be regarded as representative of the general population of women giving birth in Sweden. Participants were recruited from four urban regions and one rural region, and more than 70% had a university education, which may limit generalizability to populations with lower educational attainment or to women living in rural areas. Although the cohort originated from a randomized controlled trial, the present analyses are observational, and the randomization does not imply representativity for the current research questions. Furthermore, the definitions used to assess breastfeeding practices in this study were adapted to the national context and may therefore not be directly comparable with globally recommended breastfeeding indicators. Moreover, maternal income and other social factors, were not included in the study which could have provided additional insights into barriers and contextual challenges affecting breastfeeding duration. Also, breastfeeding at one year was assessed using a self-reported yes/no question, which may not capture variations in breastfeeding intensity or frequency and may be subject to reporting bias. In addition, we could not determine what type of formula or solids the infant was given, since the question was phrased, “How old was your child when you first fed him/her something other than breast milk?” In contrast, other studies have differentiated between infant formulas, small tastings and solids [13, 31, 32]. Therefore, we were unable to distinguish between infants who were given formulas and those who were introduced to solids, or at what age (in months) this occurred.

## Conclusion

This study highlights the importance of providing support to ensure a very positive initial breastfeeding experience, avoiding introduction of formula or solids before four months of age unless medically indicated and including social background factors. Knowledge about factors associated with breastfeeding at one year can inform healthcare practices aiming to increase breastfeeding in line with national and international recommendations. These factors should be implemented in future breastfeeding support strategies. Further research is suggested to explore breastfeeding intentions during pregnancy and a deeper understanding of women´s experiences of their first breastfeeding session.

## Data Availability

The dataset used and analyzed during the current study are available from the corresponding author upon reasonable request.

## Abbreviations

BMI: Body Mass Index
CHCN: Child Healthcare Nurse
EPDS: Edinburgh Postnatal Depression Scale
FOBS: Fear of Birth Scale
HCP: Health Care Professional
M-RCT: Multicentre Randomized Controlled Trial
NICU: Neonatal Intensive Care Unit
WBC: Well-Baby Clinic
WHO: World Health Organization
